# Smaller microorganisms outcompete larger ones in resistance and functional effects under disturbed agricultural ecosystems

**DOI:** 10.1002/imt2.219

**Published:** 2024-06-23

**Authors:** Chunling Liang, Jiejun Qi, Wenyuan Wu, Xingyu Chen, Mingyu Li, Yu Liu, Ziheng Peng, Shi Chen, Haibo Pan, Beibei Chen, Jiai Liu, Yihe Wang, Sanfeng Chen, Sen Du, Gehong Wei, Shuo Jiao

**Affiliations:** ^1^ State Key Laboratory for Crop Stress Resistance and High‐Efficiency Production, Shaanxi Key Laboratory of Agricultural and Environmental Microbiology, College of Life Sciences Northwest A&F University Yangling China; ^2^ Key Laboratory for Agrobiotechnology and College of Biological Sciences China Agricultural University Beijing China; ^3^ Fertilizer Technology Department National Agricultural Technology Extension and Service Center Beijing China

## Abstract

Body size is a key ecological trait of soil microorganisms related to their adaptation to environmental changes. In this study, we reveal that the smaller microorganisms show stronger community resistance than larger organisms in both maize and rice soil. Compared with larger organisms, smaller microorganisms have higher diversity and broader niche breadth to deploy survival strategies, because of which they are less affected by environmental selection and thus survive in complex and various kinds of environments. In addition, the strong correlation between smaller microorganisms and ecosystem functions reflects their greater metabolic flexibility and illustrates their significant roles in adaptation to continuously changing environments. This research highlights the importance of body size in maintaining stability of the soil microbiome and forecasting agroecosystem dynamics under environmental disturbances.

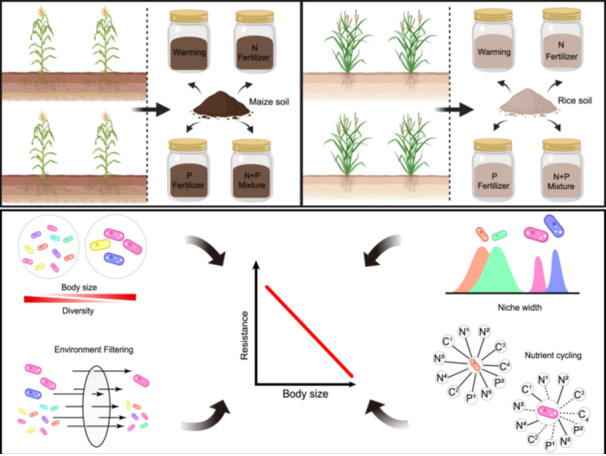

Natural and man‐made climate changes, such as climate warming and excessive use of fertilizers, seriously threaten the services and functions of agricultural ecosystems [1, 2]. The stability of biotic communities has been proven to be of great importance for maintaining multifunctionality in agricultural ecosystems [3]. Recent studies have revealed that community stability can be affected by species' life‐history traits [4]. However, we still lack a comprehensive understanding of how species life‐history traits, such as body size, influence community stability among distinct groups of soil microorganisms in agricultural ecosystems under ongoing environmental changes.

Body size, as a determinant of reproductive capacity and survival, is directly related to the organism's adaptation to environmental changes [5, 6]. Since smaller microorganisms have highly flexible metabolism [6], we hypothesize that smaller microorganisms have stronger resistance to environmental changes than larger microorganisms, and the underlying mechanisms of this phenomenon may be the result of a composite effect of many factors. Current research in microbial ecology generally reports that high species diversity is a more desirable scenario for maintaining ecosystem stability [7]. Thus, it is tempting to speculate that species‐rich communities can maintain resistance against disturbances more effectively. Furthermore, the environmental tolerance range defines an organism's niche, reflecting the community's sensibility to environmental disturbances through the scope of resources related to living conditions [8]. Microorganisms with narrow niche breadth can survive under certain conditions, whereas other taxa that can tolerate broader environmental conditions are ubiquitous with a high probability and maintain more stable species abundance [9]. Meanwhile, organisms with diverse sizes have distinct community assembly processes (deterministic and stochastic process) due to their differences in environmental adaptability. Larger organisms tend to encounter more stringent growth conditions, while smaller microorganisms are more able to withstand environmental filtering [10]. Given the significant influence of microbial communities on critical biogeochemical processes (e.g., nutrient cycling) [11], a more mechanistic understanding of the relations between differentially sized microorganisms and ecosystem functions under environmental changes is essential for accurate predictions of future ecosystem stability. Therefore, elucidating the mechanisms of community stability from multiple angles, particularly considering organisms with different body sizes, is crucial for precise management of resource‐efficient and disturbance‐resistant sustainable agroecosystems. However, empirical evidence supporting the above hypotheses and these views as explanatory factors of the community stability induced by body size is limited and lacks consensus.

Here, we considered two different crop types, including maize (dryland) and rice (wetland), to investigate the resistance and mechanism of adaptation of microorganisms with different sizes to various environmental disturbances in agricultural ecosystems. Soils collected from 50 maize and rice fields throughout eastern and southeastern areas of China were incubated to simulate expected impacts from warming and nutrient addition (nitrogen, phosphorus). Following incubation, we acquired soil bacterial, fungal, and protistan diversity information based on high‐throughput sequencing of 16S and 18S ribosomal RNA genes and identified the body size of some groups on the basis of the literature. We also measured 11 soil functions affected by soil microbial activities, which correspond to crucial ecosystem services such as carbon, nitrogen, and phosphorus cycling. Here, we aimed to address the following question: are smaller microorganisms more resistant than larger organisms in disturbed environments? Does the maintenance of ecosystem functions depend largely on the smaller microorganisms when disturbances cease?

## RESULTS AND DISCUSSION

**Figure 1:**
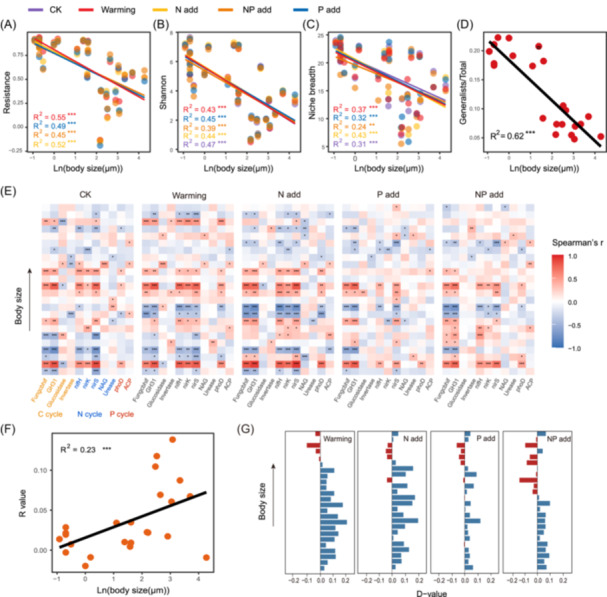
Resistance and mechanisms of adaptation of differentially sized soil microorganisms. Relationship among body size, community resistance (A), the Shannon index (B), niche breadth (C), and the proportion of generalists (D) based on linear least‐squares regression analysis. Asterisks denote significant correlations (***p < 0.001; **p < 0.01). (E) Associations between the relative abundance of single groups of 24 organisms and single ecosystem functions. Colors represent Spearman's correlations. (F) Linear regression between the logarithm of body sizes and the *R* value was obtained by calculating the correlation of differentially sized soil microorganisms and each functional factor in the control group. Asterisks denote significant correlations (***p < 0.001). (G) The *D* value (*R* value in the treated group minus *R* value in the control group) in the correlation of differentially sized soil microorganisms and each functional factor between two groups before and after the treatment.

We first determined the body size of 24 soil organism groups (12 bacterial groups, seven fungal groups, and five protistan groups) based on a previous study [12]. These groups accounted for at least more than 60% of their total sequences (Figure S1), and the body size span was quite considerable (Figure S2). Then, we estimated the resistance of differentially sized soil microorganisms under different environmental changes. The resistance was significantly and negatively related to the body sizes across 24 selected organism groups in both maize and rice soil (Figures 1A and S3A,D). Besides, the stability of differentially sized soil organism groups to environmental disturbance was further examined by evaluating the tolerance widths and the nonsynchronization of responses at the community level [13]. We observed that bacterial communities with smaller body sizes had a broader tolerance width and greater nonsynchronization of responses to disturbances (Figure S4). In combination, these findings suggested that smaller microorganisms are arguably more adaptive to environmental disturbances than larger organisms. But what makes the smaller microorganisms more resistant to environmental perturbations? Possible explanations of this observation were discussed.

It was observed that the Shannon index and niche breadth were markedly negatively correlated with body size (Figures 1B,C and S3B,C,E,F), and both were positively correlated with community resistance (Figure S5). This confirmed our first hypothesis that the higher species diversity and wider niche breadth of smaller microorganisms lead to stronger community resistance. Smaller microorganisms generally have a faster growth rate, which potentially increases the rate of mutation and evolution and results in higher diversity [14]. More coexisting species are expected to provide more buffer to protect the ecosystem against functional decline when faced with environmental changes. In addition, the niche concept is a key focus in estimating the risk of whole‐community collapse under ever‐changing environments, and communities with higher niche widths are considered to be more environmentally resistant [9].

Soil microbial generalists and specialists were identified at the amplicon sequence variants level based on the species–environment association pattern [15]. The linear fitting model showed a negative correlation between the proportion of generalists and body size (Figure 1D), which means that smaller microorganisms often tend to play the role of generalists in the agricultural ecosystem. We further quantified the relative importance of deterministic and stochastic processes under different environmental disturbances based on the neutral model and the normalized stochasticity ratio model to estimate the community assembly mechanics of differentially sized organism groups [16]. Our result showed that community stochasticity gradually decreased with increasing organism body size, that is, the smallest microorganisms (bacteria) were more regulated by stochastic processes, while larger ones (fungi and protists) were more influenced by deterministic processes (Figure S6). Therefore, smaller microorganisms mostly consisting of a high proportion of generalists can better cope with environmental filtering and mediate community stability in fluctuating environments.

Finally, we correlated differentially sized microorganisms with soil functions. Similar strong correlation patterns were observed for smaller microorganisms and ecosystem functions in all treatments (Figure 1E). In the control group, we observed that body sizes were positively correlated with the *R* value, which characterizes the strength of the link between differentially sized soil microorganisms and ecosystem multifunctionality (Figure 1F). This meant that before environmental disturbances, the smaller the body size, the weaker the relationship between soil microorganisms and ecosystem functions. After various environmental changes, we calculated the *D* value (*R* value in the treated group minus *R* value in the control group) of 24 organism groups based on two groups of *R* values. Interestingly, we found that the *D* value gradually changes from a positive value to a negative value as the body size increases (Figure 1G); this shows that the relationship between smaller microorganisms and ecosystem functions improves, but that in larger organisms, it decreases clearly. An interesting interpretation of our results is that the growth of microorganisms in samples without any treatment involved use of the limited resources in the soil with an increase in the incubation time. Under these adverse conditions, smaller microorganisms may become dormant to protect themselves [17]. Then, they may be rapidly revived by the simulated conditions of warmth and nutrient addition. Accordingly, we conclude that smaller microorganisms may have greater potential to deploy dormancy strategies, thereby ensuring their widespread and persistent survival in changing environments. Furthermore, the ability of smaller organisms to switch between active and dormant modes helps to maintain the species diversity so as to promote resistance to various environmental perturbations [17].

Notably, a relationship between body size and some response variables seems to be absent within taxonomic groups (Figures S7, S8, and S9). The limitations of collecting microbial body size data solely from the literature may yield incomplete and inaccurate results that do not consider the generic relationship between body size and response variables. It is important to note that body size varies widely among species, so some deviation in the results might be alleviated by much wider intergroup than intragroup differences [18]. Therefore, our discovery of the positive relationships among community diversity, niche breadth, and body size can be considered reliable. Of course, it is important to keep in mind that caution must be exercised when inferring ecological patterns using unverified and limited body size data. Further experimental studies and analytical research are necessary to corroborate our findings across a wider range of size gradients.

## CONCLUSION

Overall, our study demonstrates that smaller microorganisms have stronger resistance to disturbances in environments of agricultural soil and generally outcompete larger organisms. Compared with larger organisms, smaller microorganisms with high diversity have more different ecotypes (individuals occupying different niches) and have a wide niche breadth to deploy survival strategies, because of which they are less affected by environmental selection and thus survive in complex and various kinds of environments. In addition, the strong correlation between smaller microorganisms and ecosystem functions reflects greater flexibility in metabolism and its significant role in adaptation to continuously changing environments. Although further research is required to confirm the main finding outside of a control experimental scenario, it is likely that body size is a pivotal factor that affects the resistance of organisms to various environmental disturbances. Our findings could contribute to a comprehensive and in‐depth understanding of the mechanisms responsible for the stability of differentially sized soil microorganisms in agricultural ecosystems, as well as their contributions to ecosystem functioning under global environmental changes.

## AUTHOR CONTRIBUTIONS

Gehong Wei, Shuo Jiao, Chunling Liang, and Jiejun Qi conceived and designed the study. Chunling Liang, Jiejun Qi, Wenyuan Wu, Xingyu Chen, Mingyu Li, Yu Liu, Ziheng Peng, Shi Chen, Haibo Pan, Beibei Chen, Jiai Liu, and Yihe Wang collected the samples. Chunling Liang performed the experiments, analyzed the data, and wrote the manuscript. Gehong Wei, Shuo Jiao, Sanfeng Chen, Sen Du, and Jiejun Qi revised the manuscript. All authors have read the final manuscript and approved it for publication.

## CONFLICT OF INTEREST STATEMENT

The authors declare no conflict of interest.

## Supporting information


**Figure S1:** Selected species from different taxonomic groups.
**Figure S2:** Taxa and body size of selected species.
**Figure S3:** Relationships among community resistance, Shannon index, niche breadth, and body size in maize and rice soils.
**Figure S4:** Resistance of distinct organism groups to environmental changes.
**Figure S5:** Relationships among community resistance, the Shannon index, and niche breadth in maize and rice soils.
**Figure S6:** Effects of environmental disturbances on microbial community assembly processes.
**Figure S7:** Relationships among community resistance, the Shannon index, niche breadth, and body size within distinct taxonomic groups.
**Figure S8:** Relationships among the Shannon index, niche breadth, and community resistance within distinct taxonomic groups.
**Figure S9:** Relationships among the proportion of generalists, specialists, and body size within distinct taxonomic groups.

## Data Availability

The amplicon sequence data have been deposited in the Genome Sequence Archive (Genomics, Proteomics & Bioinformatics 2017) in the BIG Data Center (Nucleic Acids Research 2018), Beijing Institute of Genomics (BIG), Chinese Academy of Sciences, under accession number PRJCA010837 for the 16S data set and PRJCA010862 for the 18S data set, and are publicly accessible at: http://bigd.big.ac.cn/gsa. The data and scripts used are saved in GitHub at https://github.com/ChunlingLiang/Body-size. Supplementary Materials (methods, figures, graphical abstract, slides, videos, Chinese translated version, and updated materials) may be found in the online DOI or iMeta Science http://www.imeta.science/.
