# Supplementary material for: Smaller microorganisms outcompete larger ones in resistance and functional effects under disturbed agricultural ecosystems
Source: Imeta. 2024 Jun 23;3(4):e219. doi: 10.1002/imt2.219 (PMC11316917; doi:10.1002/imt2.219)
Supplement: Supplementary file 1 — Figure S1: Selected species from different taxonomic groups. Figure S2: Taxa and body size of selected species. Figure S3: Relationships among community resistance, Shannon index, niche breadth, and body size in maize and rice soils. Figure S4: Resistance of distinct organism groups to environmental changes. Figure S5: Relationships among community resistance, the Shannon index, and niche breadth in maize and rice soils. Figure S6: Effects of environmental disturbances on microbial community assembly processes. Figure S7: Relationships among community resistance, the Shannon index, niche breadth, and body size within distinct taxonomic groups. Figure S8: Relationships among the Shannon index, niche breadth, and community resistance within distinct taxonomic groups. Figure S9: Relationships among the proportion of generalists, specialists, and body size within distinct taxonomic groups. [file IMT2-3-e219-s001.docx]

**Supporting information to**

**Smaller microorganisms outcompete larger ones in resistance and functional effects under disturbed agricultural ecosystems**

**Running title:** Stronger resistance in smaller microorganisms than large ones

Chunling Liang^1#^, Jiejun Qi^1#^, Wenyuan Wu^1^, Xingyu Chen^1^, Mingyu Li^1^, Yu Liu^1^, Ziheng Peng^1^, Shi Chen^1^, Haibo Pan^1^, Beibei Chen^1^, Jiai Liu^1^, Yihe Wang^1^, Sanfeng Chen^2^, Sen Du^3^, Gehong Wei^1*^, Shuo Jiao^1*^

^1^State Key Laboratory for Crop Stress Resistance and High-Efficiency Production, Shaanxi Key Laboratory of Agricultural and Environmental Microbiology, College of Life Sciences, Northwest A&F University, Yangling 712100, China

^2^Key Laboratory for Agrobiotechnology and College of Biological Sciences, China Agricultural University, Beijing 100193, China

^3^National Agricultural Technology Extension and Service Center, Beijing 100125, China

^#^These authors contributed equally: Chunling Liang, Jiejun Qi

^*^ Correspondence: [weigehong@nwafu.edu.cn](mailto:weigehong@nwafu.edu.cn) (Gehong Wei); [shuojiao@nwsuaf.edu.cn](mailto:shuojiao@nwsuaf.edu.cn) (Shuo Jiao)

**MATERIALS AND METHODS**

**Soil sampling and incubation**

All samples selected to this study were agricultural soils which have a cultivation history for maize or rice at least a decade. We were successful in gathering 25 soil samples from rice and 25 samples from maize among 23 paired sites, 2 sites with only maize, and 2 sites with only rice. The maize and rice fields at each paired site were located < 5 km apart (Maize and rice fields at each paired site located within 5 km of each other). During the growing season, three 100 m^2^ plots in randomized blocks of each site were used to sample soils, and five soil cores (sampling depth is 0–15 cm) were merged into a composite sample per plot. Subsequently, these soil samples were homogenized using a 2 mm sieve in order to exclude biological fragments and pebbles.

A soil microcosm experiment was set up under laboratory conditions with the objective of simulating multiple global environmental changes. In this experiment, there are numerous treatments, which include the following: (a) control; (b) warming; (c) nitrogen (N) addition; (d) phosphorus (P) addition; and (e) combined nitrogen and phosphorus (NP) addition. Take approximately 50 g of soil samples from each site and store them in glass bottles with sealed caps, repeating each treatment three times. Also, the soils were pre-incubated in an incubator at 20°C for a week to stabilize microbial activity under the dark condition. We added sterile water to the soil of the control group to ensure that the soil water holding capacity (WHC) of the maize sample reached 60%, while making the rice sample reach a flooded state, and then incubated them at 20°C. In comparison to the control group, the warming group reached the same water content but increased in temperature (+5°C). In addition, the other three treatment groups (N added group, P added group and NP added group) were maintained without any changes in temperature and moisture content, but were separately added with 150 kg N ha^−1^ yr^−1^, 100 kg P ha^−1^ yr^−1^ and the combinations of both when compared to control group. All samples under different treatments were incubated at 20℃ in the dark room with constant conditions for 170 days. After the trail, three replicate samples were merged into one sample for the subsequent measurement of biological and abiotic indicators.

**DNA extraction and Illumina sequencing**

Total genomic DNA was extracted from soil samples via a FastDNA SPIN Kit (MP Biochemicals, Solon, OH, USA) in accordance with the manufacturer’s instructions. The diversity data of soil microbial community was obtained through high-throughput sequencing, which targets the V4-V5 region of 16S rRNA in bacteria and the V4 region of 18S rRNA in fungi and protists. The 515F/907R [1] and 528F/706R [2] primer pairs, respectively, were used to conduct corresponding polymerase chain reaction experiments. Library preparation, cluster generation, and sequencing for all amplicons were performed on the Illumina HiSeq2500 platform (Illumina Inc.). The raw sequence reads were processed in R using the DADA2 package according to a previously described pipeline. Next, we joined the paired-end reads that pass quality control steps and removed the chimeras. Taxonomy assignments of amplicon sequencing variants (ASVs) were determined on the SILVA database for bacteria and PR2 database for fungi and protists, respectively. The chloroplasts and mitochondria identified from the 16S rRNA gene sequences were removed. Due to significant differences in sequencing depth, we homogenized all the samples based on the minimum sequencing depth, resulting in 29,688 sequences for bacteria, 5,836 sequences for fungi, and 10,165 sequences for protists. We found a total of 18,648 bacterial, 8,436 fungal and 28,031 protistan ASVs in all soil samples. Twenty-four organism groups in all were selected for downstream analyses. Meanwhile,12 bacterial groups, 7 fungal groups, and 5 protistan groups accounted for more than 95%, 70%, and 60% of their total sequences, respectively (Figure S1).

**Focus taxonomic groups and body size**

One impediment to studying the relationship between body size and community stability has been the lack of precise classification and morphological description to estimate sizes for each organism. We therefore differentiated organism groups based on phylum/class level within the eukaryota and bacteria domains. The reason why we did this is that body size, as a widely defined functional trait, is usually more conservative within phyla than across phyla [3,4]. For the determination of species’ body size, propagule size appears to be more reliable compared to the mature individual size. We determined the body size of 24 soil organism group through literature [5]. Our results suggested that the body size span of 24 selected organism groups was quite considerable, with bacterial groups varied between 0.4 μm and 5 μm, fungal groups between 4.85 μm and 28.25 μm, and protistan cells between 11.88 μm and 71.89 μm (Figure S2).

**Assessing multiple ecosystem functions**

After incubation, we further measured 11 ecosystem functions and grouped them into the major ecosystem services (including five enzyme activities and eight functional genes). The functions of enzyme activity were related to sugar degradation [*β*-Glucosidase and saccharase], chitin degradation [*N*-Acetylglucosaminidase], urea hydrolysis [urease], P mineralization [phosphatase]. The extracellular enzyme in all soil samples activities were detected by fluorometry as mentioned previously [6]. The functions of functional genes were related to cellulose degradation (*Fungicbhif*), starch degradation (*GH31*), N fixation (*nifH*), denitrification (*nirK* and *nirS*) and organic P mineralization (*phoD*). Since single functions were too limited to represent the overall function of a specific ecosystem, the averaging ecosystem multifunctionality index was obtained based on these eleven ecosystem functions. Specifically, we calculated the mean of the standardized score (ranging from 0 to 1) for all individual ecosystem functions [7].

**Statistical analyses**

Along with the development of microbial ecology, multiple methods for characterizing the community resistance have gradually been proposed. Previous study showed that species composition with varying responses and tolerances to the environment determined the resistance of the communities [8]. We used community mean tolerance breadth (CMTB) and community mean response asynchrony (CMRA) to estimate the resistance potential of differentially sized soil organism groups [8]. Communities with greater CMTB or CMRA values were thought to be more resilient to environmental disturbance, with a lower probability of destroying ecosystem services driven by microorganisms. Furthermore, to quantify the sensitivity of differentially sized microorganisms to different environmental disturbance, the community resistance (Rs) was also calculated by comparing the Shannon index between each treated and control group during soil incubation experiments [9]. The formula for calculation was as follows:

$$Rs=1-\frac{2\left|D_{0}\right|}{(C_{0}+\left|D_{0}\right|)}$$

where D0 represents the difference in Shannon diversity between the control (C0) and the treated sample. RS is bounded by 1 (maximal resistance) and -1 (lowest resistance).

Shannon index values, strongly recommended, were also chosen to characterize the α-diversity of differentially sized soil microorganisms. To assess the response of microorganism to environmental filtration, we estimated niche breadth of differentially sized taxonomic groups with the “spaa” package in R [10]. According to the concept of niche breadth, we speculated that a specie with a broader niche breadth possess higher habitat availability or stronger metabolic plasticity, and are insensitive to environmental fluctuation [11,12]. We first conducted Spearman correlations to examine the relationships between the Shannon index (or niche breadth) and body size. And then, we linked the resistance of the selected differently sized organism groups to the Shannon index and niche breadth using least-squares linear regression.

The neutral community model (NCM) was used for predicting the potential effect of stochastic processes on organism groups as a method to detect the effects of environmental filtering [13]. The parameter Nm in this model is used to evaluate the dispersal ability of microorganisms, and the two letters represent population size (N) and migration rate (m), respectively. The evaluation of model fit was completed by using nonlinear least squares in “minpack” package [14]. Then we used R package of “NST” to calculate the normalized stochasticity ratio (NST) [15]. It could help us further determine the balance of the deterministic and stochastic assembly in microbial communities. We then identified specialists and generalists at ASV level and defined this threshold by comparing random and observed distributions [16]. ASVs enriched in small-scale environments were defined as specialists, while ASVs enriched in widespread environments were classified as generalists.

In order to clarify the linkage between differentially sized soil microorganisms and ecosystem functions, the general connection between ecosystem functions and soil microbial β-diversity was investigated firstly. To this end, the β-diversity of taxonomic groups were estimated using the Bray–Curtis dissimilarity index. Also, the Euclidean distance was applied to obtain the distance matrices from eleven single ecosystem functions. We then linked the β-diversity of taxonomic groups to the dissimilarity matrices from ecosystem functions using Mantel correlations. We also evaluated all possible correlations (Spearman test) between each single function and differentially sized soil microorganisms under various global environmental drivers. To gain advanced knowledge about the relationship between differentially sized organisms and ecosystem multifunctionality index, we conducted correlation analysis before and after disturbance respectively. In brief, we related organisms with different body size to the ecosystem multifunctionality index using Mantel correlations (Spearman), and then linear regression was used for modeling the connection between the extracted R value and body size. For the treated and control groups, the difference in the degrees of relevance could also be calculated by R value.

All statistical analysis and visualizations were performed in the R environment (v4.0.2; <http://www.r-project.org/>).

**REFERENCES**

1. Langenheder, Silke, Anna J. Székely. 2011. “Species sorting and neutral processes are both important during the initial assembly of bacterial communities.” *Isme Journal* 5: 1086−1094. <https://doi.org/10.1038/ismej.2010.207>

2. Zinger, Lucie, Pierre Taberlet, Heidy Schimann, Aurélie Bonin, Frédéric Boyer, Marta De Barba, Philippe Gaucher, et al. 2019. “Body size determines soil community assembly in a tropical forest.” *Molecular Ecology* 28: 528−543. <https://doi.org/10.1111/mec.14919>

3. Sexton, Jason P., Jorge Montiel, Jackie E. Shay, Molly R. Stephens, Rachel A. Slatyer. 2017. “Evolution of ecological niche breadth.” *Annual Review of Ecology, Evolution, and Systematics* 48: 183−206. https://doi.org/10.1146/annurev-ecolsys-110316-023003

4. Jiao, Shuo, Junman Wang, Gehong Wei, Weimin Chen, Yahai Lu. 2019. “Dominant role of abundant rather than rare bacterial taxa in maintaining agro-soil microbiomes under environmental disturbances.” *Chemosphere* 235: 248−259. <https://doi.org/10.1016/j.chemosphere.2019.06.174>

5. Luan, Lu, Yuji Jiang, Menghua Cheng, Francisco Dini-Andreote, Yueyu Sui, Qinsong Xu, Stefan Geisen, et al. 2020. “Organism body size structures the soil microbial and nematode community assembly at a continental and global scale.” *Nature Communications* 11: 6406. <https://doi.org/10.1038/s41467-020-20271-4>

6. Kelleher, Erin S. 2023. “Jack of all trades versus master of one: how generalist versus specialist strategies of transposable elements relate to their horizontal transfer between lineages.” *Current Opinion in Genetics & Development* 81: 102080. <https://doi.org/10.1016/j.gde.2023.102080>

7. Székely, Anna J., Mercè Berga, Silke Langenheder. 2013. “Mechanisms determining the fate of dispersed bacterial communities in new environments.” *Isme Journal* 7: 61−71. <https://doi.org/10.1038/ismej.2012.80>

8. Kuang, Jialiang, Dongmei Deng, Shun Han, Colin T Bates, Daliang Ning, Wensheng Shu, Jizhong Zhou. 2022. “Resistance potential of soil bacterial communities along a biodiversity gradient in forest ecosystems.” mLife 1: 399−411. <https://doi.org/https://doi.org/10.1002/mlf2.12042>

9. Liang, Yuting, Xian Xiao, Erin E. Nuccio, Mengting Yuan, Na Zhang, Kai Xue, Frederick M. Cohan, et al. 2020. “Differentiation strategies of soil rare and abundant microbial taxa in response to changing climatic regimes.” *Environmental Microbiology* 22: 1327−1340. <https://doi.org/10.1111/1462-2920.14945>

10. Chen, Ya J., Pok M. Leung, Jennifer L. Wood, Sean K. Bay, Philip Hugenholtz, Adam J. Kessler, Guy Shelley, et al. 2021. “Metabolic flexibility allows bacterial habitat generalists to become dominant in a frequently disturbed ecosystem.” *Isme Journal* 15: 2986−3004. <https://doi.org/10.1038/s41396-021-00988-w>

11. He, Qing, Shang Wang, Kai Feng, Sean T. Michaletz, Weiguo Hou, Wenhui Zhang, Fangru Li, et al. 2023. “High speciation rate of niche specialists in hot springs.” *Isme Journal* 17: 1303−1314. <https://doi.org/10.1038/s41396-023-01447-4>

12. Xiang, Qian, Dong Zhu, Min Qiao, Xiaoru Yang, Gang Li, Qinglin Chen, Yongguan Zhu. 2023. “Temporal dynamics of soil bacterial network regulate soil resistomes.” *Environmental Microbiology* 25: 505−514. <https://doi.org/10.1111/1462-2920.16298>

13. DeLong, John P., Jean P. Gibert, Thomas M. Luhring, Gwendolyn Bachman, Benjamin Reed, Abigail Neyer, K. L. Montooth. 2017. “The combined effects of reactant kinetics and enzyme stability explain the temperature dependence of metabolic rates.” *Ecology and Evolution* 7: 3940−3950. <https://doi.org/10.1002/ece3.2955>

14. Tabi, Andrea, Owen L. Petchey, Frank Pennekamp. 2019. “Warming reduces the effects of enrichment on stability and functioning across levels of organisation in an aquatic microbial ecosystem.” *Ecology Letters* 22: 1061−1071. <https://doi.org/10.1111/ele.13262>

15. Lennon, Jay T., Stuart E. Jones. 2011. “Microbial seed banks: the ecological and evolutionary implications of dormancy.” *Nature Reviews Microbiology* 9: 119−130. <https://doi.org/10.1038/nrmicro2504>

16. Jones, Stuart E., Jay T. Lennon. 2010. “Dormancy contributes to the maintenance of microbial diversity.” *Proceedings of the National Academy of Sciences* 107: 5881−5886. <https://doi.org/doi:10.1073/pnas.0912765107>


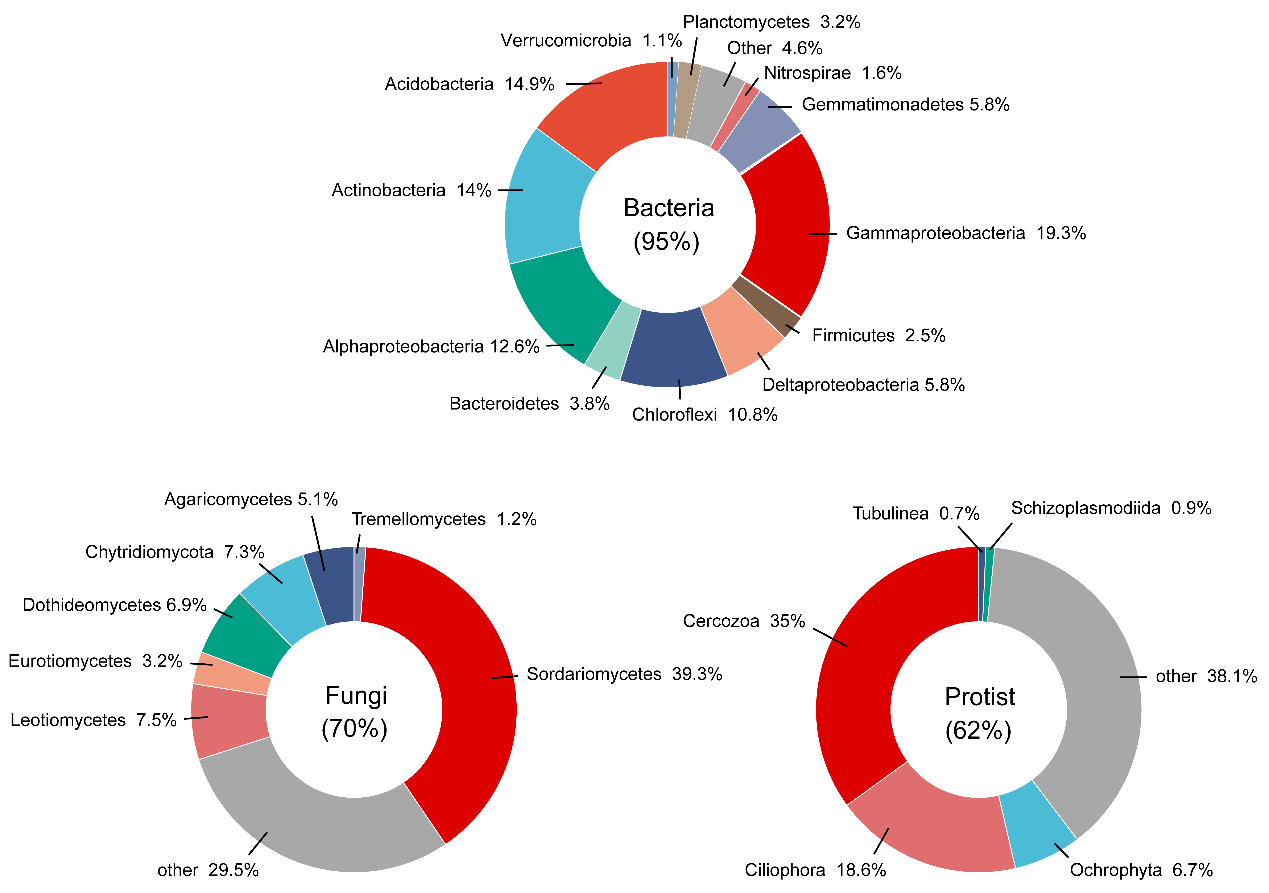


**Figure S1** **Selected species from different taxonomic groups.** Species names and the proportion of each taxa are provided outside the donut chart.


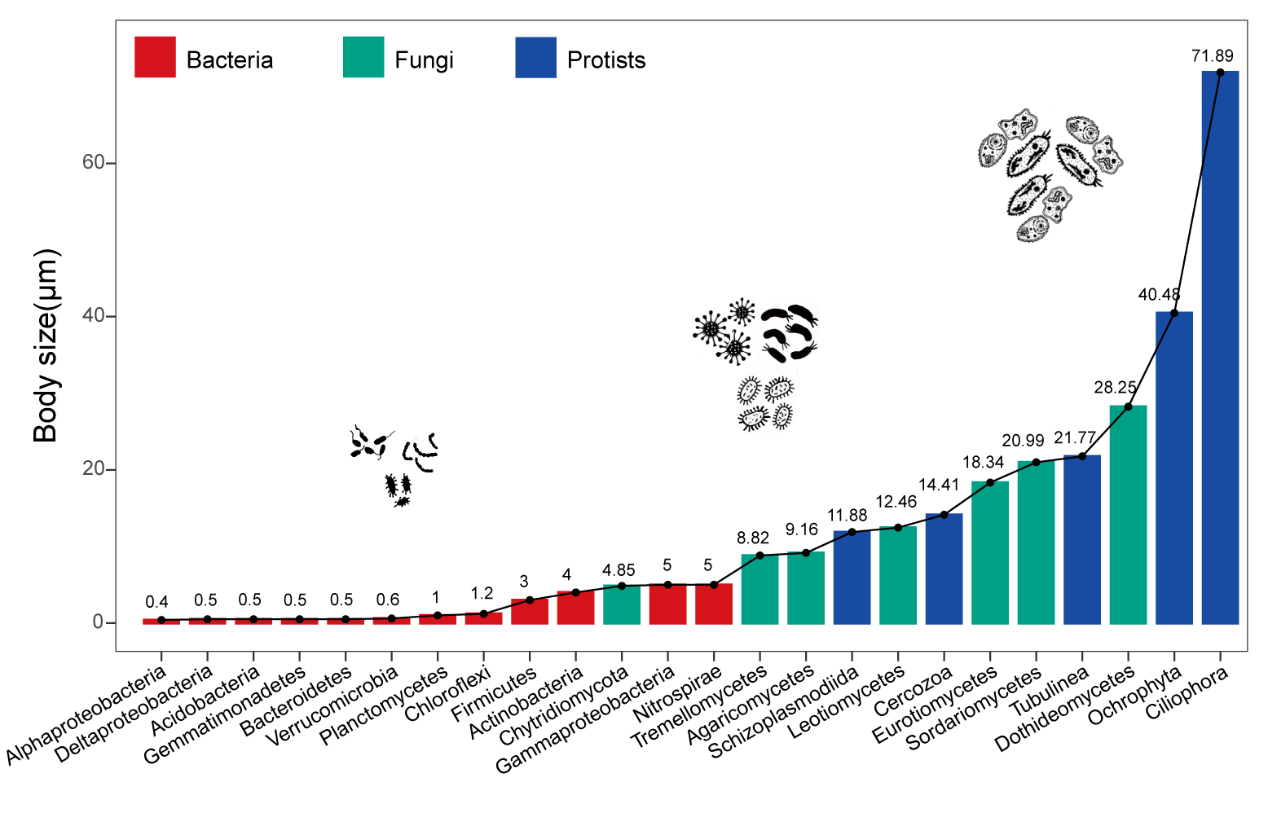


**Figure S2 Taxa and body size of selected species.** The average body size of 24 organism groups, including bacteria (12 groups), fungi (7 groups), and protists (5 groups).


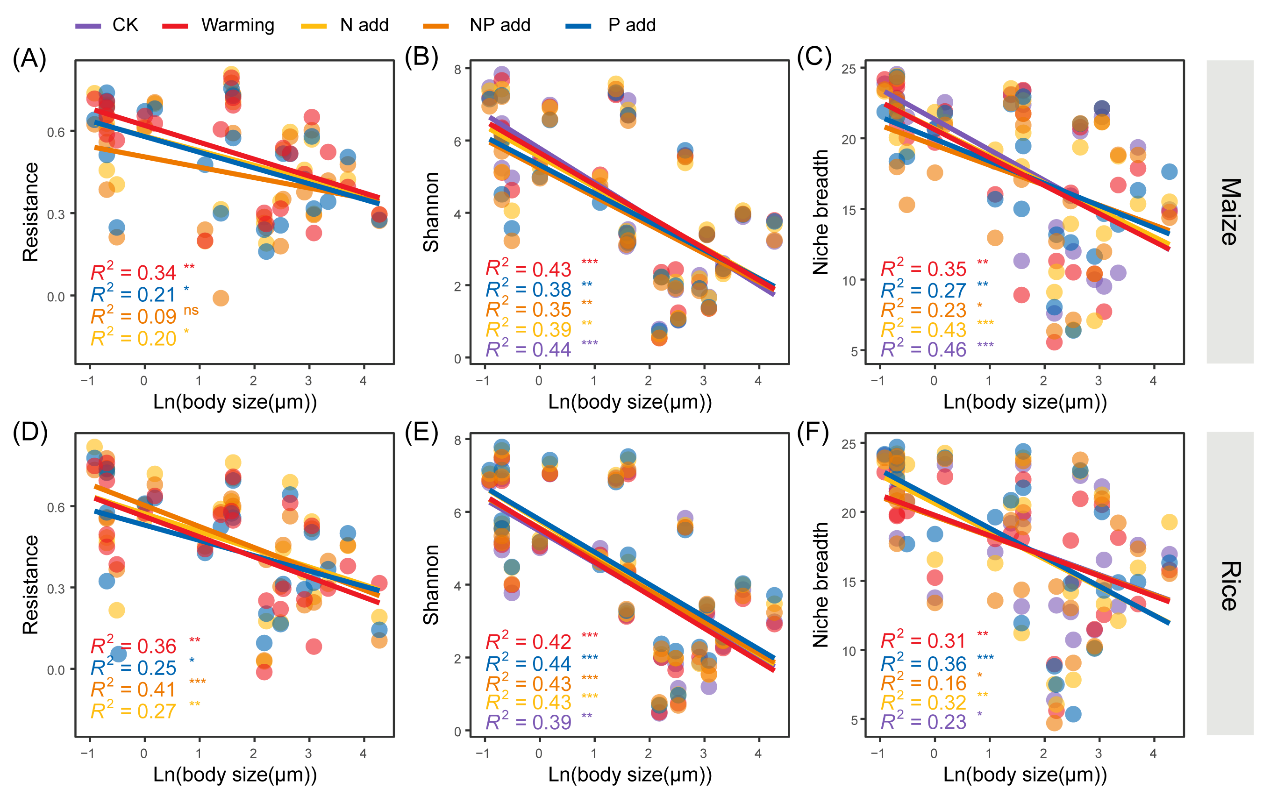


**Figure S3** **Relationships among community resistance, Shannon index, niche breadth, and body size in maize and rice soils.** Linear relationships between body size and resistance in maize (A) and rice soils (D); Linear relationships between body size and Shannon index in maize (B) and rice soils (E); Linear relationships between body size and niche breadth in maize (C) and rice soils (F); Statistical analysis was performed using ordinary least squares linear regressions. Asterisks denote significant correlation (*** *p* < .001; ** *p* < .01; * *p* < .05).


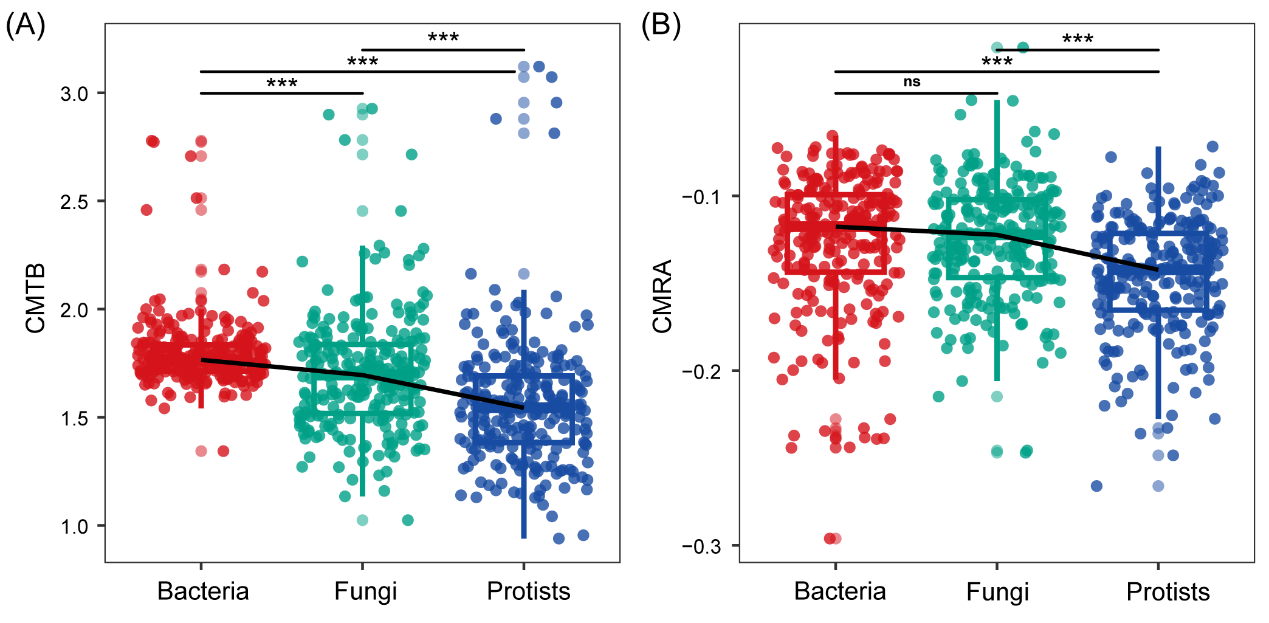


**Figure S4 Resistance of distinct organism groups under environmental changes.** Box plots showing the community mean tolerance breadth (CMTB) (A) and community mean response asynchrony (CMRA) (B) in bacterial, fungal, and protistan community (*p* values were estimated using the two-sided Wilcoxon test for pairwise comparisons; *** *p* < .001, ns indicate *p* > 0.05).


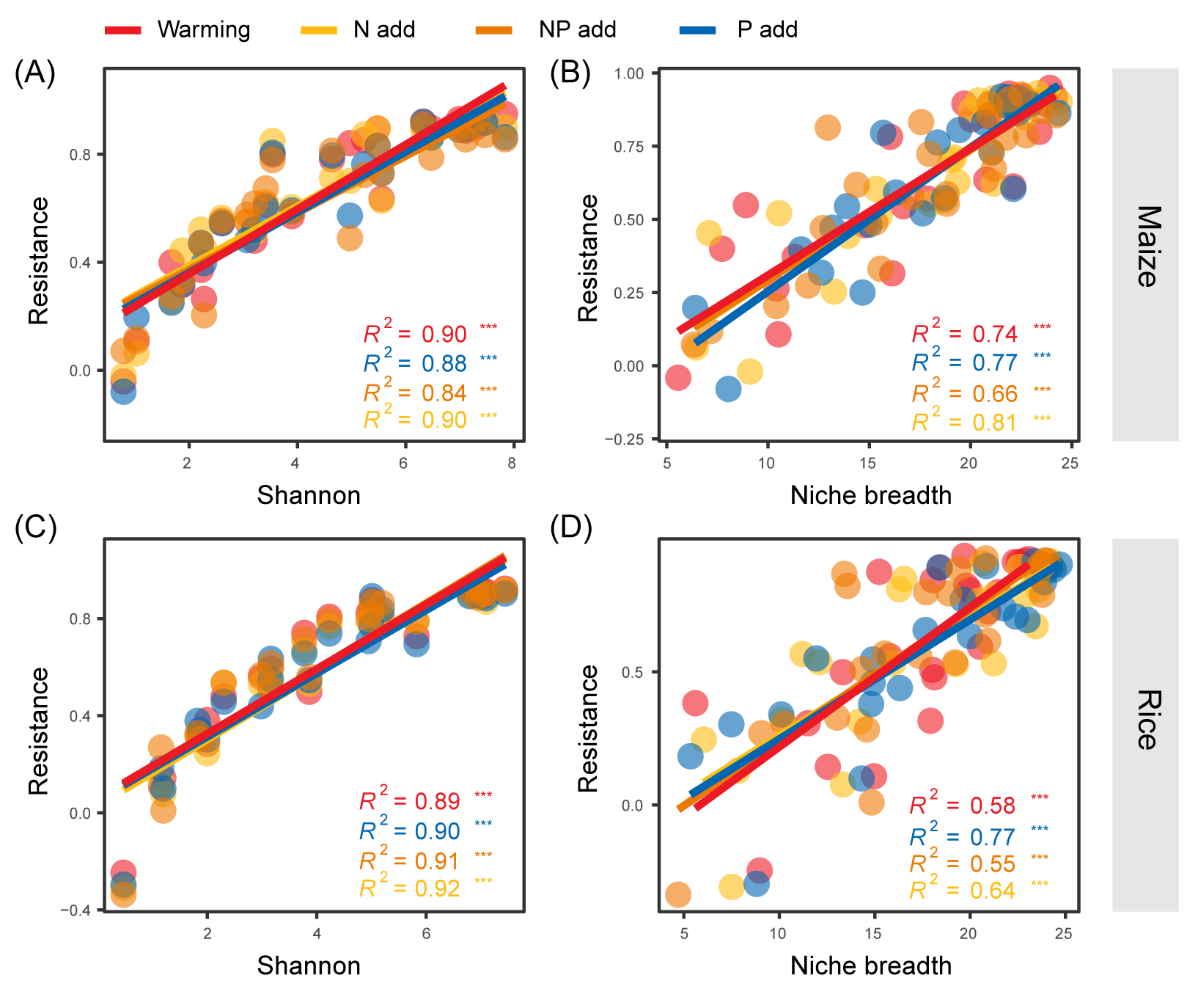


**Figure S5 Relationships among community resistance, Shannon index, and niche breadth in maize and rice soils.** Linear relationships between resistance and Shannon index in maize (A) and rice soils (C); The linear relationships between resistance and niche breadth in maize (B) and rice soils (D). Significant correlations were found using all 24 organism groups. Statistical analysis was performed using ordinary least squares linear regressions; *p* values were indicated by asterisks: ****p* < .001.


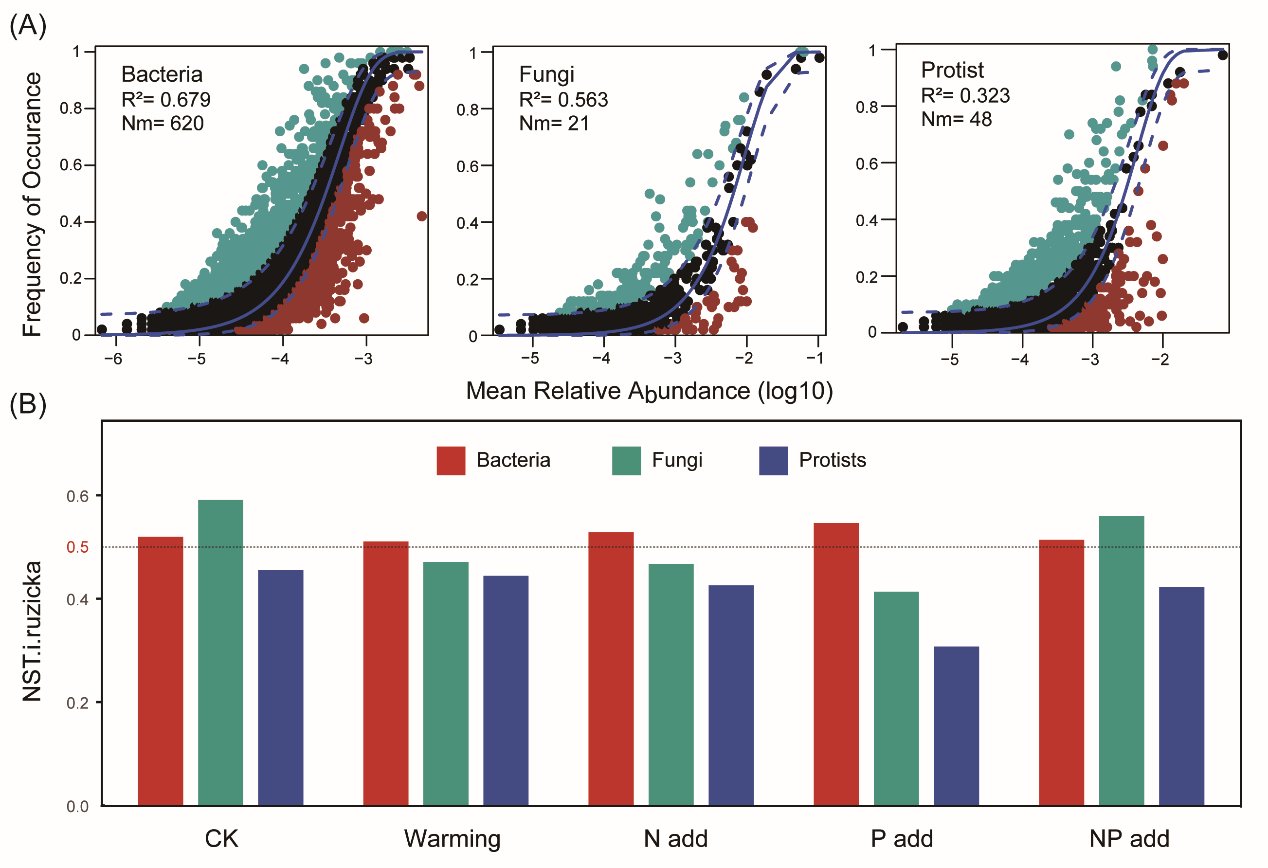


**Figure S6 Effects of environmental disturbances on microbial community assembly processes.** (A) The neutral community model (NCM) to assess soil bacterial, fungal, and protistan community assembly processes. The solid line is the best fit for the model and the dotted line is the 95% confidence intervals. ASVs that occur more or less frequently than predicted by the NCM are marked in different colors. Nm indicates the metacommunity size times immigration, R^2^ indicates the goodness of fit to this model (the higher the R^2^, the greater the goodness of fit). (B) Normalized stochasticity ratio (NST) of bacteria, fungi and protist under various conditions (control, Warming, N add, P add and NP add). Red dashed line indicates NST threshold. Values above and below the NST threshold of 50% indicate deterministic and stochastic processes, respectively.


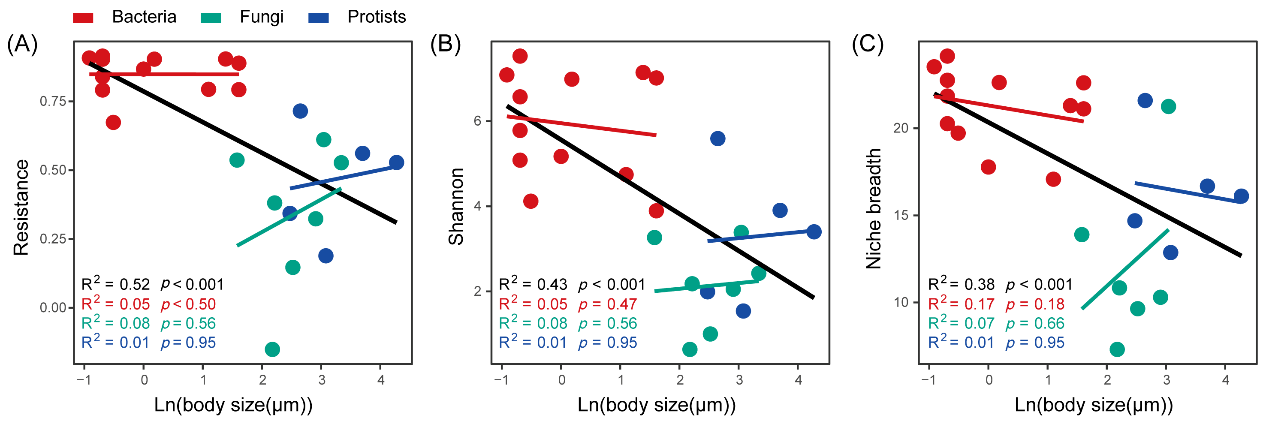


**Figure S7 Relationships among community resistance, Shannon index, niche breadth, and body size within distinct taxonomic groups.** Linear correlations between the community resistance (A), Shannon index (B), niche breadth (C), and body size for the 24 organism groups, including 12 bacterial groups, 7 fungal groups, and 5 protistan groups. We applied linear least-squares regression analysis, and the calculated *p* values as shown.


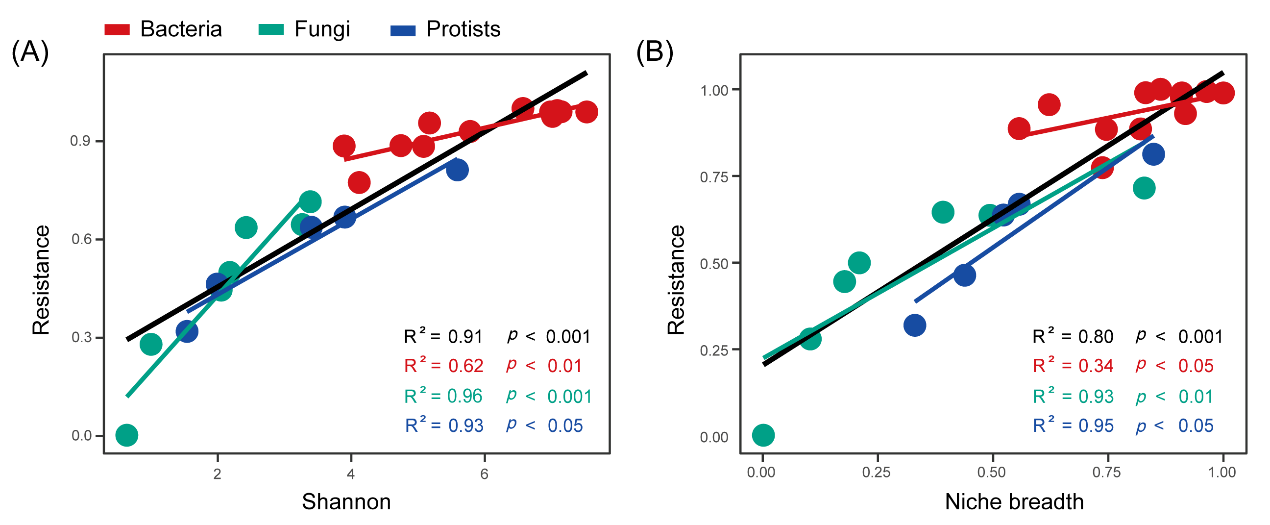


**Figure S8** **Relationships among Shannon index, niche breadth, and community resistance within distinct taxonomic groups.** Linear correlations between Shannon index (A), niche breadth (B) and the community resistance for the 24 organism groups, including 12 bacterial groups, 7 fungal groups, and 5 protistan groups. We applied linear least-squares regression analysis, and the calculated *p* values as shown.


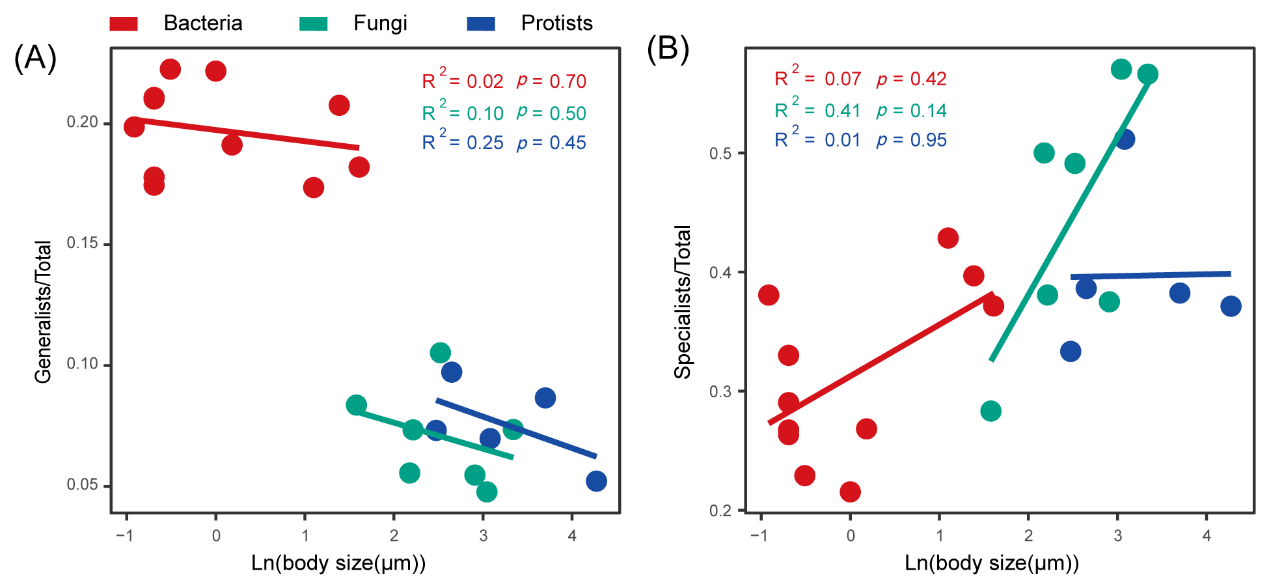


**Figure S9 Relationships among the proportion of generalists, specialists, and body size within distinct taxonomic groups.** Linear correlations between the proportion of generalists (A), specialists (B), and body size for the 24 organism groups, including 12 bacterial groups, 7 fungal groups, and 5 protistan groups. We applied linear least-squares regression analysis, and the calculated *p* values as shown.
